# Association between Lower Extremity Skeletal Muscle Mass and Impaired Cognitive Function in Type 2 Diabetes

**DOI:** 10.1038/s41598-020-59914-3

**Published:** 2020-02-19

**Authors:** Serena Low, Tze Pin Ng, Chin Leong Lim, Angela Moh, Su Fen Ang, Jiexun Wang, Kiat Sern Goh, Keven Ang, Wern Ee Tang, Pek Yee Kwan, Tavintharan Subramaniam, Chee Fang Sum, Su Chi Lim

**Affiliations:** 1 Diabetes Centre, Admiralty Medical Centre, Singapore, Block 676, Level 4, Kampung Admiralty, Woodlands Drive 71, Singapore, 730676 Singapore; 2 Clinical Research Unit, Khoo Teck Puat Hospital, 90 Yishun Central, Singapore, 768828 Singapore; 3 Gerontology Research Programme, Department of Psychological Medicine, Yong Loo Lin School of Medicine, National University of Singapore, National University Health System Tower Block, Level 9, 1E Kent Ridge Road, Singapore, 119228 Singapore; 4 Lee Kong Chian School of Medicine, Nanyang Technological University, Clinical Sciences Building, 11 Mandalay Road, Singapore, 308232 Singapore; 5 Department of Geriatrics, Changi General Hospital, Singapore, 2 Simei Street 3, Singapore, 529889 Singapore; 6 National Healthcare Group Polyclinics, Singapore, 3 Fusionopolis Link, Nexus@one-north, South Tower, Singapore, 138543 Singapore; 7 Saw Swee Hock School of Public Health, National University of Singapore, 12 Science Drive 2, #10-01, Singapore, 117549 Singapore

**Keywords:** Type 2 diabetes, Neurology

## Abstract

Lower extremity skeletal muscle mass (LESM) in Type 2 Diabetes (T2D) has been linked to adverse clinical events, but it is not known whether it is associated with cognitive difficulties. We conducted a cross-sectional study on 1,235 people (mean age 61.4 ± 8.0 years) with T2D under primary and secondary care in Singapore. Bioelectrical impedance analyses (BIA) measures of upper extremity skeletal muscle mass (UESM), LESM and appendicular skeletal muscle index (SMI) were related to the Repeatable Battery for the Assessment of Neuropsychological Status (RBANS) measures of cognition, in multiple linear regression. In multivariable models, tertile 1 LESM (b = −2.62 (−3.92 to −1.32)) and tertile 2 LESM (b = −1.73 (−2.73 to −0.73)), referenced to tertile 3) were significantly associated with decreased RBANS total score. Significant associations of LESM with cognitive domain performances were observed for tertile 1 (b = −3.75 (−5.98 to −1.52)) and tertile 2 (b = −1.98 (−3.69 to −0.27)) with immediate memory, and for tertile 1 (b = −3.05 (−4.86 to −1.24)) and tertile 2 (b = −1.87 (−3.25 to −0.48)) with delayed memory, and for tertile 1 (b = −2.99 (−5.30 to −0.68)) with visuospatial/constructional ability. Tertile 1 SMI (b = −1.94 (−3.79 to −0.08) and tertile 2 SMI (b = −1.75 (−3.14 to −0.37)) were also associated with delayed memory. There were no associations between UESM with cognitive performance. Lower LESM may be a useful marker of possible co-occuring cognitive dysfunction.

## Introduction

Sarcopenia, the age-related decline in skeletal muscle mass and strength and physical function^1,2^, is linked to adverse events and outcomes including hospitalization, disability and mortality^3–6^. Recent studies have also examined the association between sarcopenia and impaired cognitive function as both conditions are highly prevalent in advanced age^2^.

However, results have been rather divergent^7–12^ as some studies reported a link^9–11^ while others did not^7,8,12^. It is not clear if such a relationship is evident in older individuals with diabetes. Of note, older patients with long-term type 2 diabetes (T2D) typically show accelerated loss of muscle mass and strength. As skeletal muscle plays a key role in glucose storage, absorption and metabolism, diabetic myopathy is an important, but often overlooked condition, that contributes to additional complications and adverse outcomes^13^. It is plausible that sarcopenia and cognitive impairment may have a “common soil” known as age-related inflammation (i.e. “inflammaging”). Under this paradigm, environmental antigenic load accumulated over several decades may have increased the global inflammatory tone and excessive oxidative stress, thereby leading to end-organ injury like reduced cognitive function and sarcopenia^14^.

Recent studies suggest that lower extremity skeletal muscle mass (LESM) in patients with T2D is associated with sub-optimal metabolic control, which is related to obesity and cardiovascular death^15–17^. LESM, but not upper extremity skeletal muscle mass (UESM), is also reportedly associated with hospitalization risk^15^. Most of the subjects in this study were hospitalized due to hyperglycemia. This suggested that sarcopenia might be linked to sub-optimal glycemic management, and with myokines possibly as mediator. There was also speculation of reduced physical activity in subjects with lower LESM. Furthermore, lack of physical activity confers more risk of non-infectious conditions^15^.

To our knowledge, the relationship between LESM and cognitive function in persons with diabetes has not been explored. It is also not known which cognitive domains LESM is associated with. In this study, we investigated whether lower LESM in parallel with UESM and total appendicular skeletal mass index (SMI) are associated with reduced cognitive function in people with diabetes in Singapore.

## Methods

### Study subjects

This was a cross-sectional study, conducted between September 2014 and October 2017, on males and females with diabetes (>45 years old) in the Diabetes Centre in a public hospital and a public primary-care polyclinic in the north of Singapore. They were recruited as part of the Singapore Study of Macro-angiopathy and Micro-vascular Reactivity in Type 2 Diabetes (SMART2D) which aimed to study risk factors for vascular injury, diabetic complications (including renal complication) and cognitive dysfunction. These individuals were on follow-up for diabetes management at these sites. The people with diabetes in the Diabetes Centre were younger (age 60.3 ± 8.0 vs. 62.9 ± 7.9 years; p < 0.001); had longer diabetes duration (15.9 ± 9.1 vs. 13.9 ± 8.9 years; p < 0.001) compared to those in the primary-care polyclinic. Subjects were excluded if they had the following: active inflammation (e.g. systemic lupus erythromatosis or overt untreated cancer), use of non-steroidal anti-inflammatory medications on the same day of assessment, use or oral steroids equivalent to prednisolone >7.5 mg/day, contraindications to bio-impedance analysis (e.g. cardiac pacemaker), existing neuropsychiatric disease that could impede neurocognitive ability, and inability to give written informed consent.Out of 1,469 people in the cohort, the final analysis included 1,235 participants after exclusions. Written informed consent was provided by all participants. Ethics approval was given by the National Healthcare Group Domain Specific Review Board in Singapore. The methods were performed according to the relevant guidelines and regulations.

### Data collection

Trained research nurses administered a questionnaire on patient demographics, educational level, medical conditions and medications. Blood pressure (BP) was measured with a standard sphygmomanometer with patients in the siting position after resting for at least 10 minutes. Blood and urine samples were analysed at the hospital laboratory with accreditation from the College of the American Pathologists (CAP). The following clinical parameters were quantitated: Haemoglobin A1c (HbA1c) with the Tina-quant Hemoglobin A1c Gen.3 (Roche cobas® c 501); serum low density lipoprotein cholesterol (LDL-cholesterol) with enzymatic colorimetric test (Roche Cobas ®c 501); serum creatinine with enzymatic colorimetric tets (Roche cobas® c 501); and urinary albumin with immunoturbidmimetric assay (Roche Cobas ®c 501). The Chronic Kidney Disease Epidemiology Collaboration (CKD-EPI) equation formula was used to estimate glomerular filtration rate (eGFR)^18^. A 15-item Geriatric Depression Scale (GDS) was used to assess depressive symptoms. Individuals were considered as having depressive symptoms if they scored ≥5 on the GDS^19^.

### Body composition

The tetrapolar multi-frequency BIA device analysis (InBody-S10; Biospace, California, USA) was used to measure body composition. The following indices were measured and derived: waist circumference (WC), hip circumference (HC), body mass index (BMI), and skeletal muscle mass index (SMI), calculated as appendicular lean mass divided by square of height (kg/m^2^)^20^, following the recommendations of the Asian Working Group for Sarcopenia^21^. The skeletal muscle mass in the left and right upper limbs were added and divided by square of height to calculate the UESM. The skeletal mass in the left and right lower limbs were added and divided by square of height to calculate the LESM. Similarly, SMI was computed for skeletal muscle mass of the four limbs.

### Cognitive function

The Repeatable Battery for the Assessment of Neuropsychological Status (RBANS), which was translated for local application, with validated norms for Singaporean older adults^22–24^, was administered by trained nurses. RBANS assesses cognitive function in 12 subsets of tests (list learning, story memory, figure copy, line orientation, picture naming, semantic fluency, digit span, coding, list recall, list recognition, story recall and figure recall). Five domains of cognition were derived from these subsets: attention, language, visuospatial or constructional abilities, and immediate and delayed memory and an overall cognition score was derived from the five domains.

### Statistical analysis

Continuous variables were presented as means ± standard deviation (SD) or median (interquartile range), and categorical variables as number (percentage). SMI, UESM and LESM were categorised in tertiles to ensure sufficient numbers in each group especially for ethnicity and years of education. Group differences in SMI, UESM and LESM categories were examined with Chi-Square test for categorical variables, and one-way analysis of variance or Kruskal Wallis test for continuous variables as appropriate. The association between the skeletal muscle indices and RBANS scores was explored using multiple linear regression. Age, gender, ethnicity, years of education, depressive symptoms, diabetes duration, history of smoking, systolic blood pressure (SBP), LDL-cholesterol, HbA1c, BMI, body fat, urinary albumin-to-creatinine ratio, eGFR, use of metformin and use of hypolipidemics were included in the model for adjustment based on biological plausibility or P-value less than 0.10 observed in univariable linear regression. Results were deemed as statistically significant if P-values were lower than 0.05. STATA Version 14.0 (STATA Corporation, College Station, Texas) was used for statistical analysis.

## Results

The whole sample participants’ characteristics are shown in Table 1. The mean age of participants was 61.4 ± 8.0 years and there was a slight dominance of male (51.9%) over female participants (48.1%). About half of the participants were Chinese (53.8%) while Malays and Indians constituted 18.4% and 23.7% of the study population. The average duration of diabetes was 15.0 ± 9.0 years. The majority of the patients (67.5%) had at least seven years of education. The prevalence of depressive symptoms was 4.4%.

**Table 1:** Subject characteristics by lower extremity skeletal muscle mass in tertiles.

	All (N = 1235)	LESM categories			
		Tertile 1	Tertile 2	Tertile 3	P-value
Age (years)	61.4 ± 8.0	63.5 ± 8.1	61.6 ± 7.5	59.1 ± 7.9	<0.001
Male (%)	641 (51.9)	50 (12.1)	242 (58.7)	349 (84.9)	<0.001
Ethnicity Chinese	664 (53.8)	223 (54.3)	223 (54.1)	218 (53.0)	<0.001
Malay	227 (18.4)	53 (12.9)	76 (18.5)	98 (23.8)	
Indian	292 (23.7)	116 (28.2)	100 (24.3)	76 (18.5)	
Other	51 (4.1)	19 (4.6)	13 (3.2)	19 (4.6)	
DM duration (years)	15.0 ± 9.0	15.0 ± 8.9	15.5 ± 9.2	14.6 ± 9.0	0.345
Smoking: None	124 (10.1)	372 (91.4)	292 (71.1)	237 (57.8)	<0.001
Past	203 (16.5)	20 (4.9)	66 (16.1)	117 (28.5)	
Current	901 (73.4)	15 (3.7)	53 (12.9)	56 (13.7)	
Education: >10 years	289 (23.4)	54 (13.1)	87 (21.2)	148 (36.0)	<0.001
7–10 years	544 (44.1)	179 (43.5)	192 (46.7)	173 (42.1)	
1–6 years	337 (27.3)	142 (34.5)	112 (27.3)	83 (20.2)	
0 years	64 (5.2)	37 (9.0)	20 (4.9)	7 (1.7)	
Depressive symptoms (%)	54 (4.4)	24 (5.8)	15 (3.6)	15 (3.7)	0.210
Stroke (%)	68 (5.7)	20 (5.0)	26 (6.6)	22 (5.7)	0.614
SBP (mmHg)	141.9 ± 18.6	143.4 ± 18.8	140.4 ± 17.4	141.8 ± 19.4	0.067
BMI (kg/m^2^)	27.3 ± 7.5	25.1 ± 4.0	26.5 ± 4.2	30.3 ± 11.1	<0.001
WC (cm)	90.9 ± 11.8	86.3 ± 10.2	90.6 ± 10.3	95.8 ± 12.7	<0.001
HC (cm)	100.6 ± 10.3	96.3 ± 8.4	99.9 ± 9.1	105.6 ± 11.0	<0.001
Body fat mass (kg)	25.9 ± 9.5	24.1 ± 7.6	24.9 ± 9.0	28.8 ± 11.1	<0.001
Fat-free mass (kg)	44.8 ± 9.7	35.4 ± 3.6	44.6 ± 4.6	54.4 ± 8.3	<0.001
FM/FFM ratio × 10^−2^	60.0 ± 0.24	68.7 ± 0.22	57.0 ± 0.23	54.3 ± 0.23	<0.001
SMI (kg/m^2^)	9.4 ± 7.6	7.8 ± 0.7	9.1 ± 0.6	11.2 ± 12.9	<0.001
UESM (kg/m^2^)	1.8 ± 0.4	1.5 ± 0.2	1.8 ± 0.2	2.1 ± 0.4	<0.001
LESM (kg/m^2^)	5.3 ± 0.9	4.3 ± 0.3	5.2 ± 0.3	6.3 ± 0.7	<0.001
HbA1c (%)	7.8 ± 1.6	7.8 ± 1.6	7.9 ± 1.5	7.9 ± 1.6	0.627
LDL-C (mmol/l)	2.7 ± 0.8	2.6 ± 0.8	2.7 ± 0.9	2.7 ± 0.8	0.694
eGFR (ml/min/1.73 m^2^)	79.7 ± 26.9	84.5 ± 25.9	81.1 ± 25.7	73.5 ± 28.0	<0.001
UACR (mg/g)	25.0	19.0	22.3	42.7	<0.001
(IQR)	(7.1–116.)	(6.6–63.7)	(7.0–96.2)	(9.0–242.0)	
Use of metformin (%)	1015 (82.5)	347 (84.2)	338 (82.4)	330 (80.7)	0.411
Use of Hypolipidemics (%)	1082 (87.9)	361 (87.6)	362 (88.3)	359 (87.8)	0.953

DM, diabetes mellitus; SBP, systolic blood pressure; BMI, body mass index; WC, waist circumference; HC, hip circumference; FM/FFM, fat to fat-free mass; SMI, skeletal mass index; UESM, upper extremity skeletal muscle mass; LESM, lower extremity skeletal muscle mass; HbA1c, Haemoglobin A1c; FPG, fasting plasma glucose; LDL-C, low density lipoprotein cholesterol; eGFR, glomerular filtration rate; uACR, urinary albumin-to-creatinine ratio.

### LESM

When categorized by LESM in tertiles, subjects in the lowest tertile 1 tended to be older, females and received fewer years of education (p < 0.001). There were more Chinese and Indians in tertile 1 compared to tertiles 2 and 3 (p = 0.001). Compared to the higher tertile groups, they had lower BMI, WC, HC and body fat mass and SMI (p < 0.001). See Table 1.

In Table 2, comparison of neurocognitive performance scores by tertile categories of LESM showed statistically significant differences in all mean scores of RBANS total and individual cognitive domain performance (p-values < 0.001 for all and p = 0.027 for language). Notably, patients in LESM tertile 1 had lower RBANS total score, immediate memory, visuospatial/constructional, attention and delayed memory (p < 0.001).

**Table 2:** Neurocognitive performance by skeletal muscle mass measures in tertiles.

	SMI				UESM				LESM			
	Tertile 1	Tertile 2	Tertile 3	P-value	Tertile 1	Tertile 2	Tertile 3	P-value	Tertile 1	Tertile 2	Tertile 3	P-value
**RBANS**												
Total Index score	99.4 ± 7.6	100.0 ± 7.3	101.8 ± 7.0	<0.001	99.7 ± 7.4	100.2 ± 7.4	101.3 ± 7.3	0.006	99.3 ± 7.7	99.8 ± 7.1	102.0 ± 7.0	<0.001
**Domain Index scores**												
Immediate memory	99.9 ± 10.6	100.6 ± 10.4	102.5 ± 13.4	0.004	100.4 ± 10.5	100.8 ± 10.5	101.9 ± 13.4	0.142	99.7 ± 10.8	100.6 ± 10.3	102.8 ± 13.2	<0.001
Visuospatial/construction	98.4 ± 10.8	100.6 ± 14.4	102.0 ± 7.1	<0.001	98.8 ± 10.1	100.1 ± 12.5	102.0 ± 10.8	<0.001	98.4 ± 10.8	100.2 ± 12.1	102.3 ± 10.5	<0.001
Language	100.2 ± 11.0	99.6 ± 11.3	100.6 ± 11.6	0.415	100.4 ± 10.6	99.6 ± 11.7	100.3 ± 11.7	0.512	100.3 ± 11.1	99.0 ± 11.9	101.1 ± 10.9	0.027
Attention	98.7 ± 12.7	99.7 ± 12.1	102.1 ± 9.6	<0.001	98.9 ± 11.3	100.4 ± 13.5	101.2 ± 9.7	0.016	98.6 ± 12.7	99.6 ± 12.2	102.3 ± 9.4	<0.001
Delayed memory	99.6 ± 9.5	99.5 ± 8.1	101.7 ± 9.9	<0.001	99.9 ± 9.4	99.8 ± 7.8	101.0 ± 10.3	0.148	99.5 ± 9.8	99.7 ± 8.6	101.6 ± 9.1	<0.001

RBANS, repeatable battery for the assessment of neuropsychological status; MMSE, mini-mental state examination; SMI, skeletal muscle mass; UESM, upper extremity skeletal muscle mass; LESM, lower extremity skeletal muscle mass.

The regression coefficient estimates of neurocognitive performance associated with tertile categories of LESM in univariate models in Table 3 mostly persisted when adjusted for demographic and clinical characteristics in multivariate regression models in Table 4.

**Table 3:** Unadjusted coefficient (95% CI) estimates of association between skeletal muscle indices in tertiles and RBANS total and domain scores and MMSE global cognition score.

		Tertile 3	Tertile 2		Tertile 1	
RBANS Total	SMI	Reference	−1.77 (−2.77 to −0.77)	0.001	−2.42 (−3.42 to −1.42)	<0.001
	UESM	Reference	−1.12 (−2.12 to −0.12)	0.029	−1.59 (−2.60 to −0.58)	0.002
	LESM	Reference	−2.20 (−3.19 to −1.20)	<0.001	−2.72 (−3.72 to −1.73)	<0.001
Immediate	SMI	Reference	−1.91 (−3.49 to −0.34)	0.018	−2.58 (−4.16 to −1.01)	0.001
Memory	UESM	Reference	−1.13 (−2.71 to 0.46)	0.183	−1.54 (−3.13 to 0.04)	0.056
	LESM	Reference	−2.14 (−3.72 to −0.57)	0.008	−3.08 (−4.65 to −1.50)	<0.001
Visuospatial	SMI	Reference	−1.35 (−2.88 to 0.17)	0.082	−3.52 (−5.04 to −1.99)	<0.001
/Constructional	UESM	Reference	−1.87 (−3.39 to −0.34)	0.017	−3.19 (−4.72 to −1.66)	<0.001
	LESM	Reference	−2.05 (−3.57 to −0.53)	0.008	−3.87 (−5.39 to −2.34)	<0.001
Language	SMI	Reference	−1.04 (−2.59 to 0.51)	0.187	−0.44 (−1.99 to 1.11)	0.577
	UESM	Reference	−0.73 (−2.28 to 0.82)	0.357	0.12 (−1.43 to 1.67)	0.884
	LESM	Reference	−2.10 (−3.64 to −0.55)	0.008	−0.79 (−2.33 to 0.76)	0.318
Attention	SMI	Reference	−2.36 (−3.94 to −0.78)	0.003	−3.45 (−5.03 to −1.87)	<0.001
	UESM	Reference	−0.76 (−2.35 to 0.83)	0.350	−2.29 (−3.88 to −0.71)	0.005
	LESM	Reference	−2.71 (−4.29 to −1.13)	0.001	−3.73 (−5.31 to −2.15)	<0.001
Delayed	SMI	Reference	−2.18 (−3.43 to −0.92)	0.001	−2.10 (−3.36 to −0.84)	0.001
Memory	UESM	Reference	−1.14 (−2.40 to 0.13)	0.078	−1.04 (−2.30 to 0.23)	0.107
	LESM	Reference	−1.99 (−3.24 to −0.73)	0.002	−2.16 (−3.42 to −0.90)	0.001

RBANS, repeatable battery for the assessment of neuropsychological status; MMSE, mini-mental state examination; SMI, skeletal muscle mass index; UESM, upper extremity skeletal muscle mass; LESM, lower extremity skeletal muscle mass.

**Table 4:** Adjusted coefficient (95% CI) estimates of association between skeletal muscle indices in tertiles and RBANS total and domain scores MMSE global cognition score.

		Tertile 3	Tertile 2		Tertile 1	
RBANS Total	SMI	Reference	−0.96 (−1.97 to 0.04)	0.060	−1.33 (−2.67 to 0.01)	0.053
	UESM	Reference	−0.99 (−1.99 to −0.00)	0.050	−1.00 (−2.34 to 0.35)	0.147
	LESM	Reference	−1.73 (−2.73 to −0.73)	0.001	−2.62 (−3.92 to −1.32)	<0.001
Immediate	SMI	Reference	−1.21 (−2.92 to 0.50)	0.165	−1.68 (−3.97 to 0.61)	0.151
Memory	UESM	Reference	−1.26 (−2.95 to 0.44)	0.146	−1.17 (−3.47 to 1.13)	0.318
	LESM	Reference	−1.98 (−3.69 to −0.27)	0.023	−3.75 (−5.98 to −1.52)	0.001
Visuospatial	SMI	Reference	0.24 (−1.52 to 2.01)	0.143	−1.23 (−3.53 to 1.08)	0.296
/Constructional	UESM	Reference	−1.32 (−3.06 to 0.43)	0.140	−2.01 (−4.38 to 0.36)	0.097
	LESM	Reference	−1.24 (−3.01 to 0.52)	0.168	−2.99 (−5.30 to −0.68)	0.011
Language	SMI	Reference	−1.29 (−3.01 to 0.44)	0.143	−1.23 (−3.53 to 1.08)	0.296
	UESM	Reference	−1.34 (−3.04 to 0.37)	0.124	−1.28 (−3.59 to 1.04)	0.279
	LESM	Reference	−2.13 (−3.85 to −0.40)	0.016	−2.01 (−4.26 to 0.24)	0.080
Attention	SMI	Reference	−0.81 (−2.54 to 0.92)	0.358	−0.47 (−2.78 to 1.85)	0.692
	UESM	Reference	0.20 (−1.51 to 1.92)	0.816	0.41 (−1.92 to 2.73)	0.730
	LESM	Reference	−1.44 (−3.17 to 0.29)	0.104	−1.32 (−3.59 to 0.94)	0.251
Delayed	SMI	Reference	−1.75 (−3.14 to −0.37)	0.013	−1.94 (−3.79 to −0.08)	0.041
Memory	UESM	Reference	−1.27 (−2.64 to 0.11)	0.070	−0.93 (−2.79 to 0.93)	0.328
	LESM	Reference	−1.87 (−3.25 to −0.48)	0.008	−3.05 (−4.86 to −1.24)	0.001

Adjusted for age, gender and ethnicity, years of education, depressive symptoms, dm duration, history of stroke, smoking, systolic blood pressure, LDL-cholesterol, HbA1c, body mass index, estimated glomerular filtration rate and log-transformed urinary albumin-to-creatinine ratio, body fat, use of metformin and use of hypolipidemic treatment.

RBANS, repeatable battery for the assessment of neuropsychological status; SMI, skeletal muscle mass index; UESM, upper extremity skeletal muscle mass; LESM, lower extremity skeletal muscle mass.

LESM tertile 1 (b = −2.62 (−3.92 to −1.32)), and LESM tertile 2 (b = −1.73 (−2.73 to −0.73), (referenced to tertile 3) were significantly associated with decreased RBANS total score. Significant associations of lower LESM with lower cognitive domain performances were observed for LESM tertile 1 (b = −3.75 (−5.98 to −1.52)) and tertile 2 (b = −1.98 (−3.69 to −0.27)) with immediate memory, and for LESM tertile 1 (b = −3.05 (−4.86 to −1.24)) and tertile 2 (b = −1.87 (−3.25 to −0.48)) with delayed memory, for LESM tertile 1 (b = −2.99 (−5.30 to −0.68)) with visuospatial/constructional ability, and for LESM tertile 2 (b = −2.13 (−3.85 to −0.40)) with language.

### UESM and SMI

The data for UESM and SMI tertile categories are presented in the Supplementary Table 1, and show similar patterns of relationships with most demographic and clinical variables, but with fewer cognitive performance variables. In Table 2, similar patterns of associations between these skeletal muscle indices and cognitive function as LESM but with fewer significant results were observed.

Although the regression coefficient estimates of RBANS total scores were associated for tertile categories of UESM and SMI in the univariate model in Table 3, the statistical significance was lost in fully adjusted model in Table 4. In Table 4, there were no associations between UESM with cognitive performance. SMI tertile 1 (b = −1.94 (−3.79 to −0.08)) and SMI tertile 2 (b = −1.75 (−3.14 to −0.37)) were only associated with delayed memory.

## Discussion

In our study, lower LESM, but not UESM,was independently associated with reduced cognitive function globally and specifically in domains of immediate memory, delayed memory and visuospatial/constructional ability. On the other hand, SMI was associated only with delayed memory and lower UESM was not associated with cognitive function.

A small growing body of population studies which examined the association between sarcopenia and cognitive impairment have shown divergent results^7–12^. We identified three studies which supported a positive relationship^9–11^, and three which did not^7,8,12^. Positive associations were reported only when studies used sarcopenia definitions which incorporated muscles mass (measured by DEXA or BIA) and strength or gait speed. Negative results were observed when examining the specific association of low muscle mass alone with cognitive impairment, suggesting that muscle mass contributed little or not at all to explain the association of sarcopenia with impaired cognition. It is worth noting that all the above-mentioned studies involved community-living older persons. Our study is notable for demonstrating a positive association of low muscle mass alone with impaired cognitive function in a group of persons with diabetes. To our knowledge, there are no other studies of persons with diabetes that have demonstrated the link between low muscle mass and impaired cognition. There was earlier report of reduced muscle strength in the lower extremities but not in upper extremities in T2D. Such distribution of muscle strength in the extremities might be reflective of neuropathy specific to T2D^25^. It was also not known previously if there was any difference between upper and lower limb skeletal muscle mass in their relationship with cognition and what domains were involved in the relationship. Interestingly, lower limb muscle mass loss, and not upper limb muscle mass loss, appears to be specifically associated with impaired cognition in our study. Also, the positive association was observed for global cognition and specifically for immediate and delayed memory and visuospatial/constructional ability, and probably language, but not for attention. Further research is needed to elucidate the pathophysiological mechanism accounting for the relationships pertaining to different domains.

Proximal lower limb muscle loss is a prominent clinical indicator of sarcopenia in patients with diabetes and heart failure. Prior studies have highlighted the prognostic significance of a small thigh circumference for an increased risk of developing heart disease and total mortality^11^. BIA measure of reduced lower limb muscle mass was shown to be associated with increased risk of the metabolic syndrome and hospitalization in patients with type 2 diabetes^10^ and also, no association for upper limb muscle mass was shown. Despite being a component of sarcopenia, UESM may lack the fine measurement accuracy that lower limb muscle mass has. Given the mechanical stressor (e.g. weight bearing vs. non-weight bearing) on lower and upper limbs muscle may differ systematically^26^, we postulate that their myocytes biology may also differ, thereby contributing to the differential relationship with cognitive function.

Interestingly, a specific association between lower SMI and lower score for delayed memory was observed. The amount of the skeletal muscle mass, with a larger contribution from lower limb muscle mass, may play an important role in pathophysiological mechanism of delayed memory. Further studies are needed to elucidate the mechanism.

In persons with diabetes, the inability to preserve muscle mass (and function) is attributed to insulin resistance, sub-optimal glycemic management, adipose tissue deposition in skeletal muscles, and diabetic neuropathy^15,26^. Diabetes (and pre-diabetes) are well established to be associated with cognitive impairment, cognitive decline and dementia^27,28^. The shared pathophysiological mechanisms and pathways linking low muscle mass and function to cognitive impairment include insulin signaling^6,10,29,30^, hormonal^2^ and vascular dysregulations, oxidative stress and chronic inflammation^9^. Given the critical roles of insulin signalling on muscle anabolism and catabolism, receptor-binding and phosphorylation, insulin resistance strongly potentiates a sarcopenic state, through increased protein degradation via the activated ubiquitin-proteasome pathway and inhibiting the mammalian target of the rapamycin pathway^31^. For example, in the study by Wang X *et al*., the proteasome chymotryptic-like peptidase from gastrocnemius muscles in db/db mice was significantly higher than wild-type littermates which acted as controls. This demonstrated that the enhanced muscle protelolysis was linked to UPP activation in db/db mice^32^. It was noted that dysregulated insulin signalling pathways also play a key role in the pathogenesis of Alzheimer’s disease by disrupting brain processing of Abeta precursor protein and amyloid clearance and increased activation of glycogen synthase kinase 3B (GSK-3B) generating phosphorylated tau, in parallel with cerebral microvascular changes responsible for vascular dementia^33^. Although it is not the intention of this manuscript to ascertain the presence of dementia and its subtype (e.g. Alzheimer’s disease or vascular dementia) among the participants, the mechanistic link between LESM and diminished cognitive function may have patho-biology in common with that of major forms of dementia such as Alzheimer’s disease or vascular dementia.

Physical inactivity resulting from sarcopenia may also decrease the expression of insulin-like growth factor-1 (IGF-1) and brain-derived neurotrophic factor (BDNF) which are known to be modulating factors for brain neuroplasticity^10^. It has been reported that physical activity confers neuroprotective effects by activating BDNF and IGF-1, lowering susceptibility to vascular diseases and improving cerebral blood flow, and inhibiting inflammation and oxidative stress^10,34^. For example, elderly women who underwent a 3-month physical activity intervention experienced increase in serum BDNF levels during the first and third months of the programme compared to those without exercise (p < 0.01)^34^. Therefore, further research is needed to explore the role of BNDF in the interplay of physical activity, lower extremity skeletal muscle mass and cognitive function.

The temporal relationship between muscle mass and cognitive function cannot be established in this cross-sectional study. Longitudinal studies of community-living older persons indicate a co-temporal relationship whereby physical pre-frailty/frailty precedes the onset of cognitive impairment or decline and dementia over 3 to 10 years^35–37^. Cognitive impairment alone has also been shown in a fewer number of studies to precede the onset of pre-frailty/frailty^37,38^. Future studies should follow up persons with diabetes to establish whether baseline muscle mass predict subsequent risk of cognitive impairment or decline or whether sarcopenia and reduced cognitive function are both outcomes of the same pathophysiological processes.

Our study also characterized the multiple cognitive domains that were associated with low muscle mass in the lower limbs. Specifically, low muscle mass in the lower limbswas robustly associated with immediate memory, delayed memory, and visuospatial/constructional ability. Studies of community-dwelling older adults have observed that certain domains – perceptual motor function, language and memory – were consistently associated with frailty across studies^39–41^. A recent systematic review found that cross-sectional studies consistently reported that the metabolic syndrome was most consistently associated with reduced performance on executive function tasks, whereas associations with performance in attention/working memory/information processing, memory, language, and construction/motor performance domains were mixed^42^. Our results on the cognitive domains of immediate memory, delayed memory, and visuospatial/constructional ability highlighted the need for closer attention in specific areas of diabetes self-care or management such as medication adherence, self-monitoring of blood glucose and appointment keeping.

We observed that the association between LESM with cognitive function persisted after adjusting for age, gender and ethnicity, years of education, depressive symptoms, diabetes duration, stroke, smoking, systolic blood pressure, LDL-cholesterol, HbA1c, body mass index, estimated glomerular filtration rate and log-transformed urinary albumin-to-creatinine ratio, body fat, use of metformin and use of hypolipidemic treatment. This suggests that the link between muscle mass and cognitive function was independent of these metabolic factors. However, other factors such as physical and psychosocial activity that contribute to cognitive reserve were not measured in this study. There is a need to examine the role of physical activity in future studies.

There are a few clinical implications from our study. Firstly, LESM measurement may potentially be a useful “marker” of possible co-occuring cognitive dysfunction, thereby facilitating earlier detection and management. Our novel finding will provide impetus for a longitudinal study to establish the causality of the relationship between LESM and reduced cognitive function. Lastly, the association between LESM and specific cognitive domains on immediate memory, delayed memory, and visuospatial/constructional ability highlights the need to focus on aspects of diabetes self-care and management which require the use of such domains.

Skeletal muscle mass was assessed using BIA, which has been shown to have good correlation with DEXA and magnetic resonance imaging^43,44^. The reliability of BIA is well established and it is commonly used in many studies^44,45^ including previous studies involving this cohort^46,47^. There were no measurements of skeletal muscle strength or physical performance measures in this study. It is thus remarkable that low muscle mass alone was found to be associated with cognitive impairment among patients with type 2 diabetes in this study, but this findings should not be generalized to community dwelling older persons without diabetes. Furthermore, we did not exclude participants based on factors which may contribute directly or indirectly to decrease in muscle mass such as diabetic foot, orthopedic disorders and status post-stroke). Although we adjusted for history of stroke which occurred in 5.7% of our study population, we were unable to adjust for the other factors such as diabetic foot and orthopedic disorders due to lack of information on these conditions. The lack of data on physical activity in our study also precludes the examination of its role in the relationship between LESM and cognitive function.

## Conclusion

Lower LESM, not UESM, in people with type 2 diabetes is associated with poorer cognitive function, particularly immediate and delayed memory and visuospatial/construction. SMI is associated only with cognitive domain of delayed memory. Lower LESM may be a useful marker of possible co-occuring cognitive dysfunction.

## Supplementary information

Supplementary Table 1.

## Data Availability

The datasets generated and/or analysed during the current study are available from the corresponding author on reasonable request.
