# Supplementary material for: Association between Lower Extremity Skeletal Muscle Mass and Impaired Cognitive Function in Type 2 Diabetes
Source: Sci Rep. 2020 Feb 19;10:2956. doi: 10.1038/s41598-020-59914-3 (PMC7031513; doi:10.1038/s41598-020-59914-3)
Supplement: Supplementary file 1 — Supplementary Table 1. [file 41598_2020_59914_MOESM1_ESM.docx]

**Association between Lower Extremity Skeletal Muscle Mass and Impaired Cognitive Function in Type 2 Diabetes**

Serena Low^1,2^, Tze Pin Ng^3^, Chin Leong Lim^4^, Angela Moh^2^, Su Fen Ang^2^, Jiexun Wang^2^, Kiat Sern Goh^5^, Keven Ang^2^, Wern Ee Tang^6^, Pek Yee Kwan^6^, Tavintharan Subramaniam^1^, Chee Fang Sum^1^, Su Chi Lim^1,2,7^

1. Diabetes Centre, Admiralty Medical Centre, Singapore

Block 676, Level 4, Kampung Admiralty, Woodlands Drive 71, Singapore 730676

1. Clinical Research Unit, Khoo Teck Puat Hospital, Singapore

90 Yishun Central, Singapore 768828

1. Gerontology Research Programme, Department of Psychological Medicine,

Yong Loo Lin School of Medicine, National University of Singapore

National University Health System Tower Block, Level 9,

1E Kent Ridge Road, Singapore 119228

1. Lee Kong Chian School of Medicine, Nanyang Technological University

Clinical Sciences Building, 11 Mandalay Road, Singapore 308232

1. Department of Geriatrics, Changi General Hospital, Singapore

2 Simei Street 3, Singapore 529889

1. National Healthcare Group Polyclinics, Singapore

3 Fusionopolis Link, Nexus@one-north, South Tower, Singapore 138543

1. Saw Swee Hock School of Public Health, National University of Singapore

12 Science Drive 2, #10-01, Singapore 117549

Corresponding Author

Associate Professor Lim Su Chi

Diabetes Centre, Admiralty Medical Centre,

676 Woodlands Drive 71,

#03-01 Kampung Admiralty

Singapore 730676.

Telephone: +65 6602353

Fax Number: +65 66023772

Email address: [lim.su.chi@ktph.com.sg](mailto:lim.su.chi@ktph.com.sg)

**SUPPLEMENTARY TABLE**

| **Supplementary Table 1** Subjectcharacteristics by skeletal muscle mass index and upper extremity skeletal muscle mass by tertile categories | | | | | | | | | |  |
| --- | --- | --- | --- | --- | --- | --- | --- | --- | --- | --- |
|  | **SMI** | | | |  | **UESM** | | | |  |
|  | **Tertile 1** | **Tertile 2** | **Tertile 3** | **P-value** |  | **Tertile 1** | **Tertile 2** | **Tertile 3** | **P-value** |  |
|  |  |  |  |  |  |  |  |  |  |  |
| Age (years) | 64.3 ± 8.1 | 61.6 ± 7.4 | 58.3 ± 7.4 | <0.001 |  | 64.3 ± 8.0 | 61.0 ± 7.8 | 58.9 ± 7.4 | <0.001 |  |
| Male (%) | 62 (15.1) | 229 (55.6) | 350 (85.2) | <0.001 |  | 63 (15.3) | 245 (59.5) | 333 (81.0) | <0.001 |  |
| Ethnicity Chinese | 238 (57.9) | 227 (55.1) | 199 (48.4) | <0.001 |  | 249 (60.6) | 228 (55.3) | 187 (45.5) | <0.001 |  |
| Malay | 49 (11.9) | 74 (18.0) | 104 (25.3) |  |  | 50 (12.2) | 71 (17.2) | 106 (25.8) |  |  |
| Indian | 109 (26.5) | 96 (23.3) | 87 (21.2) |  |  | 100 (24.3) | 98 (23.8) | 94 (22.9) |  |  |
| Other | 15 (3.7) | 15 (3.6) | 21 (5.1) |  |  | 12 (2.9) | 15 (3.6) | 24 (5.8) |  |  |
| DM duration (years) | 15.2 ± 8.8 | 16.0 ± 9.6 | 14.0 ± 8.6 | 0.007 |  | 15.3 ± 8.9 | 15.3 ± 9.3 | 14.6 ± 8.9 | 0.461 |  |
| Smoking: None | 373 (91.4) | 299 (73.1) | 229 (55.7) | <0.001 |  | 378 (92.7) | 295 (72.3) | 228 (55.5) | <0.001 |  |
| Past | 22 (5.4) | 63 (15.4) | 118 (28.7) |  |  | 20 (4.9) | 65 (15.9) | 118 (28.7) |  |  |
| Current | 13 (3.2) | 47 (11.5) | 64 (15.6) |  |  | 10 (2.5) | 49 (12.0) | 65 (15.8) |  |  |
| Education: >10 years | 54 (13.1) | 89 (21.7) | 146 (35.5) | <0.001 |  | 62 (15.1) | 98 (23.8) | 129 (31.4) | <0.001 |  |
| 7-10 years | 180 (43.7) | 191 (46.5) | 173 (42.1) |  |  | 182 (44.2) | 190 (46.2) | 172 (41.9) |  |  |
| 1-6 years | 144 (35.0) | 108 (26.3) | 85 (20.7) |  |  | 133 (32.3) | 104 (25.3) | 100 (24.3) |  |  |
| 0 years | 34 (8.3) | 23 (5.6) | 7 (1.7) |  |  | 35 (8.5) | 19 (4.6) | 10 (2.4) |  |  |
| Depressive symptoms (%) | 23 (5.6) | 17 (4.2) | 14 (3.4) | 0.303 |  | 23 (5.6) | 17 (4.2) | 14 (3.4) | 0.303 |  |
| Stroke (%) | 22 (5.5) | 22 (5.5) | 24 (6.1) | 0.907 |  | 17 (4.3) | 26 (6.5) | 25 (6.5) | 0.295 |  |
| SBP (mmHg) | 143.9 ± 19.3 | 140.8 ± 17.0 | 140.9 ± 19.3 | 0.025 |  | 143.6 ± 18.9 | 142.5 ± 19.2 | 139.5 ± 17.5 | 0.005 |  |
| BMI (kg/m^2^) | 24.4 ± 3.5 | 27.2 ± 4.2 | 30.3 ± 11.1 | <0.001 |  | 24.3 ± 3.6 | 26.8 ± 3.8 | 30.8 ± 11.0 | <0.001 |  |
| WC (cm) | 84.6 ± 9.3 | 91.6 ± 10.2 | 96.5 ± 12.5 | <0.001 |  | 83.0 ± 8.3 | 90.5 ± 9.1 | 99.2 ± 11.6 | <0.001 |  |
| HC (cm) | 95.6 ± 7.6 | 100.8 ± 9.6 | 105.4 ± 11.1 | <0.001 |  | 95.2 ± 7.4 | 100.4 ± 8.8 | 106.2 ± 11.3 | <0.001 |  |
| Body fat mass (kg) | 23.2 ± 7.2 | 26.2 ± 8.9 | 28.5 ± 11.3 | <0.001 |  | 22.6 ± 7.1 | 25.4 ± 8.5 | 29.9 ± 11.1 | <0.001 |  |
| Fat-free mass (kg) | 35.6 ± 3.8 | 44.3 ± 4.9 | 54.4 ± 8.1 | <0.001 |  | 35.8 ± 4.2 | 45.1 ± 5.8 | 53.4 ± 8.8 | <0.001 |  |
| FM/FFM ratio x 10^-2^ | 66.0 ± 0.22 | 60.5 ± 0.24 | 53.5 ± 0.23 | <0.001 |  | 64.1 ± 0.22 | 58.1 ± 0.24 | 57.8 ± 0.25 | <0.001 |  |
| SMI (kg/m^2^) | 7.7 ± 0.6 | 9.1 ± 0.4 | 11.3 ± 12.9 | <0.001 |  | 7.8 ± 0.7 | 9.2 ± 0.6 | 11.2 ± 12.9 | <0.001 |  |
| UESM (kg/m^2^) | 1.4 ± 0.2 | 1.8 ± 0.2 | 2.2 ± 0.4 | <0.001 |  | 1.4 ± 0.2 | 1.8 ± 0.1 | 2.2 ± 0.4 | <0.001 |  |
| LESM (kg/m^2^) | 4.4 ± 0.4 | 5.2 ± 0.4 | 6.2 ± 0.7 | <0.001 |  | 4.4 ±0.6 | 5.3 ± 0.7 | 6.0 ± 0.7 | <0.001 |  |
| HbA1c (%) | 7.7 ± 1.5 | 8.0 ± 1.6 | 7.8 ± 1.5 | 0.049 |  | 7.7 ± 1.5 | 8.0 ± 1.7 | 7.8 ± 1.4 | 0.034 |  |
| LDL-C (mmol/l) | 2.6 ± 0.8 | 2.7 ± 0.9 | 2.7 ± 0.8 | 0.354 |  | 2.6 ± 0.8 | 2.7 ± 0.9 | 2.7 ± 0.8 | 0.052 |  |
| eGFR (ml/min/1.73m^2^) | 83.2 ± 26.3 | 79.7 ± 26.9 | 76.3 ± 27.2 | 0.001 |  | 83.1 ± 26.0 | 78.5 ± 28.2 | 77.5 ± 26.2 | 0.007 |  |
| UACR (mg/g) | 20.0 | 23.3 | 39.3 | <0.001 |  | 19.9 | 24.8 | 37.8 | 0.001 |  |
| (IQR) | (6.3-67.0) | (7.0-120.6) | (8.2-190.0) |  |  | (7.0-67.0) | (6.6-123.7) | (9.0-182.5) |  |  |
| Use of metformin (%) | 342 (83.2) | 333 (81.0) | 340 (83.1) | 0.646 |  | 344 (83.7) | 329 (80.1) | 342 (83.6) | 0.291 |  |
| Use of Hypolipidemics (%) | 364 (88.6) | 354 (86.1) | 364 (89.0) | 0.398 |  | 362 (88.1) | 358 (87.1) | 362 (88.5) | 0.819 |  |
|  |  |  |  |  |  |  |  |  |  |  |

SMI, skeletal muscle mass index; UESM, upper extremity skeletal muscle mass
